# Sirtuins at the Service of Healthy Longevity

**DOI:** 10.3389/fphys.2021.724506

**Published:** 2021-11-25

**Authors:** Mateusz Watroba, Dariusz Szukiewicz

**Affiliations:** Department of Biophysics, Physiology and Pathophysiology, Faculty of Health Sciences, Medical University of Warsaw, Warsaw, Poland

**Keywords:** sirtuins, anti-aging mechanisms, caloric restriction, neurodegeneration prevention, metabolic austerity, anti-inflammatory action, protective effects

## Abstract

Sirtuins may counteract at least six hallmarks of organismal aging: neurodegeneration, chronic but ineffective inflammatory response, metabolic syndrome, DNA damage, genome instability, and cancer incidence. Moreover, caloric restriction is believed to slow down aging by boosting the activity of some sirtuins through activating adenosine monophosphate-activated protein kinase (AMPK), thus raising the level of intracellular nicotinamide adenine dinucleotide (NAD^+^) by stimulating NAD^+^ biosynthesis. Sirtuins and their downstream effectors induce intracellular signaling pathways related to a moderate caloric restriction within cells, mitigating reactive oxygen species (ROS) production, cell senescence phenotype (CSP) induction, and apoptosis as forms of the cellular stress response. Instead, it can promote DNA damage repair and survival of cells with normal, completely functional phenotypes. In this review, we discuss mechanisms of sirtuins action toward cell-conserving phenotype associated with intracellular signaling pathways related to moderate caloric restriction, as well as some tissue-specific functions of sirtuins, especially in the central nervous system, heart muscle, skeletal muscles, liver, kidneys, white adipose tissue, hematopoietic system, and immune system. In this context, we discuss the possibility of new therapeutic approaches.

## Introduction

Sirtuins took their name from Sir2 (silent information regulator 2), NAD^+^ dependent histone deacetylase which has been shown to slow down replicative aging in yeast. Increased Sir2 activity extends yeast replicative life span (Sinclair and Guarente, 1997; Guarente, 2000). Sirtuins attracted some attention of researchers when they presumed that inducing sirtuin action may be responsible, or at least co-responsible, for lifespan-extending effects of caloric restriction (Guarente, 2000; Tissenbaum and Guarente, 2001; Rogina and Helfand, 2004; Bordone and Guarente, 2005). In mammals, there are seven orthologs of yeast Sir2, called sirtuins (SIRT1 – SIRT7). Even if not all of them are used to silence transcription, they all have a common molecular mechanism of substrate targeting, through deacetylation, deacylation, or O-ADP-ribosylation, using NAD^+^ as a co-substrate. Mammalian sirtuins can have a different subcellular location, chemical structure, or target proteins (Michan and Sinclair, 2007), yet they are activated mainly by caloric restriction, i.e., the same way as yeast Sir2.

## Caloric Restriction, Sirtuins, and Caloric Restriction-Induced Intracellular Signaling Pathways

Caloric restriction results in a decreased influx of glucose and free fatty acids (FFA) into the cells, slowing down the tricarboxylic acid cycle (TCA). Since many TCA reactions are coupled with transforming NAD^+^ to NADH, the mitochondrial NAD^+^/NADH ratio can be increased by caloric restriction and decreased by a high-calorie diet. Increased concentration of NAD^+^, as a co-substrate for sirtuins, can be sufficient to boost their activity (Xiao et al., 2018). Caloric restriction can decrease intracellular ATP concentration, at the same time increasing AMP concentration. A 20 to 30% increase of the AMP/ATP ratio can improve the efficiency of oxidative phosphorylation because, in such circumstances, oxidative phosphorylation does not undergo end-product inhibition by ATP, which could be possible if intracellular ATP level were already high. Increased AMP concentration may activate sirtuins indirectly through stimulating NAD^+^ biosynthesis through inducing AMPK. A high-calorie diet results in the opposite effects: accelerating the rate of TCA reactions, decreased NAD^+^/NADH ratio and increased ATP concentration, because of abundance of substrates that can be used for its production. When ATP concentration is already high, and TCA is accelerated at the same time, there is a fall of NAD^+^/NADH ratio in the mitochondria, accompanied by an excess of free electrons, delivered by NADH, in the oxidative phosphorylation cascade. When intracellular ATP concentration is high, the oxidative phosphorylation cascade may become deranged, so some free electrons provided by NADH do not find acceptors, resulting in their non-enzymatic transfer to oxygen atoms, resulting in the production of reactive oxygen species (ROS) (Yeong et al., 2008; Murphy, 2009; Mailloux, 2015).

The premises mentioned above account for at least two metabolic pathways regulating sirtuin activity: one initiated by a change in AMP/ATP ratio through AMPK; and another induced by a change in NAD^+^/NADH ratio. Improved availability of NAD^+^ can activate all sirtuins, while increased AMP concentration can activate all sirtuins except SIRT4 (Guarente, 2011).

The starting point for research studies on the role of sirtuins in the biology of aging was discovering the activating role of AMP in reference to AMPK and, at the same time, the activating role of AMPK concerning most sirtuins. At that stage of the research studies, sirtuins were believed to be the missing link between caloric restriction and life span extension (Haigis and Sinclair, 2010; Burnett et al., 2011). From today’s perspective, it can be stated that even if sirtuin activation is not the only mechanism by which caloric restriction can extend life span, it may certainly be one of the mechanisms responsible, including increased NAD^+^/NADH ratio in the mitochondria (initiated by reduced glucose influx to the cells) and decreased mTOR activity in lysosomes (also directly linked to moderate deficiency of energetic substrates within the cells) (Papadopoli et al., 2019).

Applying caloric restriction, through reducing food availability up to 60% of what would be ingested if food were available *ad libitum*, results in moderate cellular undernutrition, which in turn facilitates the following effects: reduced ATP/AMP ratio due to increased AMP concentration; activation of AMP-activated kinase (AMPK); inhibition of mTOR activity; enhanced autophagy; and induction of caloric restriction-related intracellular signaling pathways in the cell, in part due to activation of the mechanisms mentioned above (Yeong et al., 2008; Guarente, 2011; Xiao et al., 2018; Ki and Hae, 2019).

Induction of caloric restriction-induced intracellular signaling pathways in the context of some aspects of cell behavior – such as cell auto-conservation (improved fidelity of translation, DNA damage repair, and protein turnover), cell cycle arrest, and anabolism inhibition can be in part due to SIRT1-dependent deacetylation of some vital regulatory proteins (e.g., FoxO1, FoxO3a, and p53). These actions can, in turn, change the enzymatic profile of these proteins through post-translational regulatory modifications (PTRMs), altering their affinity to individual substrates. And thus – general direction of their action, which subsequently results in an altered cell reaction to metabolic and genotoxic stress – e.g., a preference to repair DNA damage instead of inducing apoptosis, or to perform reversible cell cycle arrest instead of inducing cell senescence phenotype (CSP – comprising an irreversible cell cycle arrest, loss of cytokinesis, thus excluding the cell from the processes of tissue regeneration and remodeling) (Brunet et al., 2004). For example, such alterations in cell phenotype result from FoxO3a deacetylation by SIRT1 and subsequent Gadd-45 activation by deacetylated FoxO3a (Erol, 2010; Hori et al., 2013; Salvador et al., 2013). In general, cells undergoing caloric restriction care much more about DNA damage repair, cell cycle control, translational fidelity, and proteostasis, which in turn makes them much more resistant to environmental insults. Furthermore, this shift in cell behavior results directly from improved NAD^+^/NADH ratio and resulting sirtuins activation, as well as sirtuin-dependent secondary effects within intracellular signaling (Weidinger et al., 2008; Erol, 2010; Hori et al., 2013).

SIRT1 exerts anti-inflammatory and anti-lipogenic actions through inhibitory deacetylation of NF-KB and inhibition of SREBP-1c (Imai and Guarente, 2010; Ponugoti et al., 2010; Yang et al., 2011). In addition, SIRT1 actions that promote cell auto-conservation and induce caloric restriction-related cell phenotype are exerted through FoxO1 and FoxO3a activation and co-activation of SIRT6, which can be generated through the joined action of SIRT1, FoxO3a, and NRF-1 transcription factor – also induced by caloric restriction (Gertler and Cohen, 2013).

While the coexistence of overnutrition or insulin-related intracellular signaling pathways with DNA damage will usually generate either apoptosis or CSP, DNA damage coexisting with moderate undernutrition will rather lead to gradual removal of the damaged biomolecules through autophagy. In such case, CSP will not be induced, but rather replaced with a reversible cell cycle arrest. Therefore, the cell can return to its normal metabolic functions after the damage has been completely repaired (Brunet et al., 2004; Weidinger et al., 2008; Erol, 2010; Hori et al., 2013; Salvador et al., 2013).

**FIGURE 1:**
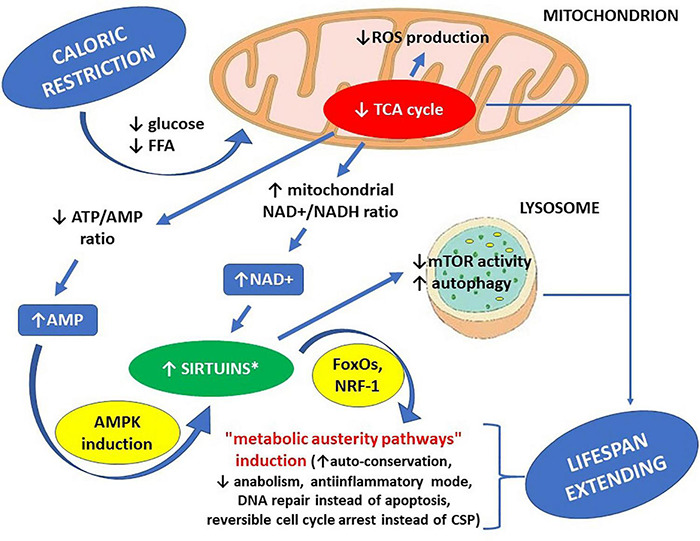
Life span-extending effects of caloric restriction – the key role of sirtuins. ^∗^ increased availability of NAD+ can activate all sirtuins, while increased AMP concentration can activate all sirtuins except SIRT4. AMP – adenosine monophosphate, AMPK – AMP-activated protein kinase, ATP – adenosine triphosphate, CSP – cell senescence phenotype, FFA – free fatty acids, FoxOs – forkhead box proteins, mTOR – mammalian target of rapamycin kinase (a protein), NAD+ – nicotinamide adenine dinucleotide, NADH – nicotinamide adenine dinucleotide hydride (reduced NAD+), NRF-1 – nuclear respiratory factor 1 (a transcription factor), ROS – reactive oxygen species, TCA cycle – tricarboxylic acid cycle.

**FIGURE 2:**
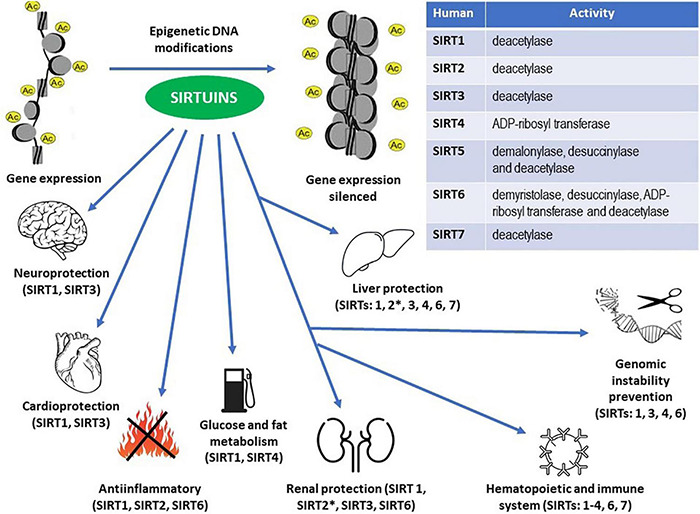
Enzymatic activities within the sirtuin family members (Table) and epigenetic DNA modifications resulting in the proven protective effects throughout the human body (see the main text for details). ^∗^ Unlike other sirtuins, SIRT2 may exert pro-inflammatory actions in the kidneys and pro- inflammatory and pro-fibrotic effects in the liver.

These same actions of sirtuins may be co-responsible for the lifespan-extending effects of caloric restriction (Erol, 2010; Gertler and Cohen, 2013; Hori et al., 2013; Salvador et al., 2013). General depiction of caloric restriction effects toward sirtuin actions is presented on Figure 1, while both general profile and general effects of sirtuin enzymatic activity within human cells are shown on Figure 2.

## How Sirtuins Prevent Neurodegeneration

The most neuroprotective sirtuins comprise SIRT1 and SIRT3 (Min et al., 2010; Jiang et al., 2011; Donmez et al., 2012; Rangarajan et al., 2015). In case of SIRT1, its neuroprotective actions are associated chiefly with its effect on some transcription factors, while SIRT3 is associated with its antioxidative and mitochondria-protective actions (Rangarajan et al., 2015). SIRT1 induction in neurons results in mTOR inhibition, activation of ADAM-10 transcription factor, and increased biosynthesis of chaperone proteins. Furthermore, the inhibitory effect of SIRT1 on mTOR activity results in promoting neurite outgrowth. In contrast, the remaining effects of SIRT1 in neurons depend on decreased production and increased degradation of toxic protein aggregates, like beta-amyloid, directly responsible for neurodegeneration. Besides, SIRT1 is neuroprotective in reference to POMC-ergic neurons responsible for adjusting energy expenditure to body nutritional status (Ramadori et al., 2008, 2010; Clark et al., 2012). Therefore, SIRT1 KO mice are more susceptible to diet-induced obesity due to insufficient energy expenditure (Ramadori et al., 2010).

Furthermore, research studies on transgenic BRASTO mice suggest that even central nervous system-confined SIRT1 overexpression tends to increase their lifespan due to local inactivation of NF-κB, and thus desensitizing the hypothalamus to TNF-alpha dependent signaling (Satoh et al., 2013; Zhang G. et al., 2013). On the other hand, neuroprotective actions of SIRT3 have not been studied as thoroughly so far. Still, we know that SIRT3 KO in microglial cells results in elevated mitochondrial ROS production, accompanied by reduced MnSOD activity (Rangarajan et al., 2015). Thus, the neuroprotective actions of SIRT3 may be dependent on its antioxidative and mitochondria-protecting effects.

## Sirtuins in Heart Muscle Cells

### The Role of Sirtuins in the Case of Cardiac Ischemia

Cardiac ischemia results in a rapid ATP depletion in heart muscle cells that change their metabolic profile and thus use a broader spectrum of substances to obtain energy to survive in such conditions (Yamamoto and Shinmura, 2021). Long-lasting ischemia can release cytochrome C from mitochondria, which in turn can induce heart muscle cell apoptosis. In addition, opening the mitochondrial permeability transition pores (mPTP) may cause an abnormal rise of intracellular calcium concentration, ATP depletion, and fall of intracellular pH, which, in turn, can induce heart muscle cell necrosis (Majno and Joris, 1995). In such conditions, reperfusion may improve the supply of oxygen and nutrients needed for ATP production and remove some toxic metabolites released from necrotic cells. Still, when following ischemia, it can promote ROS production, both by neutrophils – due to neutrophil activation by some biomolecules damaged during the preceding ischemia, and by other cells – due to hypoxanthine accumulation resulting from increased ATP degradation as a result of the preceding ischemia. Hypoxanthine accumulation combined with reperfusion-associated rise of tissue molecular oxygen concentration can make the enzyme xanthine dehydrogenase act in reverse, that is as a xanthine oxidase which may convert molecular oxygen into highly reactive superoxide and hydroxyl radicals. The phenomena mentioned above are responsible for reperfusion injury, consisting of molecular oxygen use for ROS production rather than for normal cellular respiration during reperfusion (Eltzschig and Eckle, 2011).

After reperfusion, oxidative damage related to ROS excess may impair the functioning of both mitochondria and endoplasmic reticulum (ER), leading to an irreversible remodeling of heart muscle if it becomes a chronic process. Such remodeling of the heart muscle may gradually cause ventricular dilation and impaired contractility.

SIRT1 expression falls in heart muscle cells during ischemia, while in SIRT1-overexpressing mice, heart muscle condition is more rapidly brought back to normal after ischemia, which can be related to SIRT1-mediated activation of MnSOD, Trx-1, and Bcl-xL, as well as SIRT1-dependent inhibition of Bax protein, mediated by FoxOs (Hsu et al., 2010). SIRT1 activation with resveratrol alleviates ischemic damage to the heart by promoting two anti-apoptotic effects – ERK phosphorylation, as well as p38 and JNK inhibition (Becatti et al., 2012). Applying an NAD^+^ booster, NMN also protects the heart muscle from the detrimental effects of ischemia by providing more NAD^+^ as a co-substrate for sirtuins (Yamamoto et al., 2014). Cardioprotective effects of SIRT1 can also be mediated by deacetylation of p53, thus reducing its pro-apoptotic activity (Matsushima and Sadoshima, 2015), and by deacetylation of eIF2-alpha protein, which protects cardiomyocytes from ER stress-induced apoptosis (Prola et al., 2017).

SIRT3 may also exert a cardioprotective effect by improving overall efficacy of electron transport chain (ETC) through deacetylating complex I and complex III, thus reducing production of ROS as oxidative phosphorylation byproducts, as well as improving free fatty acid use for ATP production (Banz and Rieben, 2012; Gorsuch et al., 2012). SIRT3 expression falls in ischemic heart muscle (Lombard et al., 2007), while its overexpression protects the heart through MnSOD activation (Ahn et al., 2008). Another cardioprotective action of SIRT3 may occur through deacetylation of cyclophilin D (CypD) – a regulatory component of mPTP – at lysine 166, which prevents mitochondrial damage from increased permeability of the mitochondrial membrane, and thus helps to avoid age-related congestive heart failure (Hafner et al., 2010; Porter et al., 2014; Klishadi et al., 2015). The renin-angiotensin-aldosterone pathway may be co-responsible for ischemic cardiac damage (Baker et al., 1992; Bochaton et al., 2015), and since angiotensin II inhibits SIRT3 (Baker et al., 1992), cardioprotective action of ACE inhibitors and angiotensin II receptor blockers can be, at least in part, related to rescuing SIRT3 function (Yang et al., 1997; Lombard et al., 2007; Gorsuch et al., 2012; Yamamoto et al., 2020).

### The Role of Sirtuins in Cardiac Hypertrophy

Moderate (five-fold) overexpression of SIRT1 in mice protects them from age-related cardiac hypertrophy, but a pronounced (12-fold) overexpression may enhance the hypertrophy (Alcendor et al., 2007) through deacetylation of PH domain of Akt kinase and PDK1 protein, enhancing Akt-dependent intracellular signaling, promoting mitosis and cardiomyocyte proliferation which can directly lead to cardiac hypertrophy (Sundaresan et al., 2011). These results indicate that the direction of SIRT1 actions toward heart muscle can depend on the cause of cardiac hypertrophy and the quantity of SIRT1.

SIRT3 prevents cardiac hypertrophy through FoxO3 activation, with subsequent induction of MnSOD, resulting in reduced intracellular ROS concentration, thus suppressing ROS-induced mitogen-activated protein kinases which could otherwise directly promote hypertrophy, such as Ras and Akt/PI3K (Sundaresan et al., 2009). SIRT3 expression in the course of cardiac hypertrophy rises in moderate hypertrophy but falls in pronounced hypertrophy, so it behaves differently than the expression of SIRT1 (Sundaresan et al., 2008). Overexpression of SIRT3 protects mice from phenylephrine-induced cardiac hypertrophy, while SIRT3 KO acts in the opposite direction. In addition, SIRT3 deacetylates and activates NMNAT3 – an enzyme supporting NAD^+^ synthesis (Nikiforov et al., 2011). Increased activity of this enzyme can improve mitochondrial NAD^+^ availability for SIRT3, enhancing the anti-hypertrophic actions of SIRT3 (Yue et al., 2016; Tang et al., 2017).

## Sirtuins in the Regulation of Inflammatory Response

Chronic but ineffective inflammation (inflammaging) takes part in the pathogenesis of many age-related diseases. SIRT1, SIRT2, and SIRT6 inhibit inflammatory response through inactivation of NF-κB, with detailed means of inactivation discussed further (Vazquez et al., 2021). NF-κB is a transcription factor that stimulates pro-inflammatory phenotype-related genes during some types of the cellular stress response. NF-κB is composed of five subunits: p50, p52, p65 (RelA), RelB, and c-Rel. During NF-κB activation, p50/p65, p50/c-Rel, or p52/RelB dimers are relocated to the cell nucleus (Vazquez et al., 2021).

NF-κB activation may occur through the canonical or non-canonical pathway. Still, in standard conditions, the canonical path is blocked by default due to IkB proteins, which sequestrate NF-κB in the cytoplasm. However, pro-inflammatory stimuli may activate IkB kinase (IKK), which promotes IkB degradation through inhibitory phosphorylation, and thus relocation of NF-κB to the cell nucleus (Vazquez et al., 2021). Sirtuins may inhibit NF-κB both directly and indirectly. Firstly – SIRT1 and SIRT2 can deacetylate NF-κB’s p65 subunit at lysine 310, which directly inhibits NF-κB activity (Yeung et al., 2004; Rothgiesser et al., 2010). Furthermore, such acetylation impedes methylation of adjacent lysine residues (K314 and K315), promoting ubiquitination and degradation of p65 (Rothgiesser et al., 2010; Yang et al., 2010). Secondly – SIRT1 can inhibit NF-κB through inhibitory phosphorylation of its transcriptional activators, such as PARP-1 and p300 histone acetyltransferase (Bouras et al., 2005; Rajamohan et al., 2009). Thirdly – SIRT1 and SIRT6 may inhibit the expression of NF-κB target genes due to transcriptional silencing through H3K9 DAC (Bouras et al., 2005). SIRT6 can induce the production of IkB at the level of transcription, which exerts an anti-inflammatory effect because IkB blocks the canonical pathway of NF-κB activation by default (Kawahara et al., 2009). In addition, SIRT6 may both desensitize cells to TNF-alpha, an upstream inducer of NF-κB, and inhibit TNF-alpha secretion. SIRT1 and SIRT6 actions described above are primarily responsible for their anti-inflammatory effects.

In BRASTO mice, increased expression of SIRT1 in the central nervous system can augment their lifespan and desensitize the hypothalamus to TNF-alpha (Zhang G. et al., 2013). In this way, “anti-inflammatory” action at the level of the hypothalamus produces an anti-aging and lifespan-extending effect.

## Sirtuins in the Prevention of Metabolic Syndrome, Through Acting on Skeletal Muscles, Adipose Tissue, and Liver, Among Others

Both sirtuins and their co-substrate NAD^+^ can prevent pathomechanisms of type 2 diabetes mellitus (T2DM) (Yoshino et al., 2011). While SIRT1 enhances pancreatic beta cells response to hyperglycemia in insulin secretion (Bordone et al., 2005), SIRT4 may blunt this response (Ahuja et al., 2007). However, SIRT4 activity is inhibited by caloric restriction. Therefore, caloric restriction promotes the appropriate response of pancreatic beta cells to hyperglycemia by inhibiting SIRT4 and activating SIRT1. In this way, it may improve glycemic parameters in metabolic syndrome or T2DM and even prevent those diseases. In addition, by acting on white adipose tissue, SIRT1 promotes the production of adiponectin – a protein that can prevent insulin resistance (Banks et al., 2008).

A separate matter is an effect of sirtuins toward lipolysis and gluconeogenesis in hepatocytes, adipocytes, and skeletal muscle cells, through the PGC-1 alpha transcription factor (Heilbronn et al., 2007). By activating PGC-1 alpha, SIRT1 can prevent metabolic syndrome by inducing lipolysis and enhancing glucose uptake with GLUT4 transporter. These actions of PGC-1 alpha are associated with its role as a transcription factor activated in case of cell undernutrition. In addition, sirtuins improve peripheral tissues sensitivity to insulin by inactivating PTPT1B phosphatase (Sun et al., 2007), which could otherwise weaken insulin-dependent signaling through dephosphorylation of IRS-1 and IRS-2 tyrosine residues (Seely et al., 1996; Goldstein et al., 2000; Zhang, 2007).

## Actions of Sirtuins in the Kidneys

### Anti-apoptotic Actions

SIRT1, SIRT3, and SIRT6 exert an anti-apoptotic action toward mesangial cells, podocytes, and tubular epithelial cells, observed during experiments with inducing several kinds of experimental nephropathies in mice (Perico and Benigni, 2021). SIRT1 deacetylates p53 (Kume et al., 2006) and SMAD7 (at lysine residues 60 and 70). The latter modification results in the weakening of TGF-beta dependent proapoptotic signaling (Kume et al., 2007). In addition, SIRT1 deacetylates FoxO4, which inhibits the expression of Bim proapoptotic protein (Chuang et al., 2011). SIRT3 can prevent hypertensive nephropathy and angiotensin II-induced renal fibrosis in mice through activatory deacetylation of KLF15 protein (Li N. et al., 2017). SIRT6 exerts an anti-apoptotic action toward podocytes by inhibition of Notch signaling through transcriptional silencing of *Notch1* and *Notch4* genes because of H3K9 deacetylation, as well as by preventing actin filaments derangement, and thus podocyte foot process effacement and detachment (Liu et al., 2017).

The meaning of the actions described above is strictly related to experimental contexts in which those actions have been observed and includes: ameliorating the course of diabetic nephropathy (actions on TGF-beta signaling), alleviating nephrotoxic actions of *cis*-platin (actions on p53), and prevention of experimental obstructive nephropathy in mice, induced by unilateral ureter obstruction (He et al., 2010; Kim D. H. et al., 2011; Ren et al., 2017).

### Anti-inflammatory Actions

SIRT1 and SIRT3 exert anti-inflammatory actions in the kidneys. SIRT1 can exert such effect through deacetylation of p65 subunit of NF-κB (Kitada et al., 2011; Kauppinen et al., 2013), while SIRT3 may exert anti-inflammatory effect through acting on NRLP3 inflammasome, reducing ROS concentration and pro-inflammatory cytokine production (Yang et al., 2016; Zhao et al., 2016; Zhang J. et al., 2017).

SIRT2 may exert pro-inflammatory actions in the kidneys because LPS-induced kidney injury in SIRT2 KO mice has a less severe course than in wild type mice (Jung et al., 2015).

### Antifibrotic Actions

SIRT1, SIRT3, and SIRT6 can exert antifibrotic actions in the kidneys. SIRT1 may achieve this effect by weakening TGF-beta dependent signaling through deacetylation of SMAD3 and SMAD4 molecules, which inhibits collagen production, fibronectin, and MMP7 (He et al., 2010; Li et al., 2010; Simic et al., 2013; Loboda et al., 2016; Zhang Y. et al., 2017). In addition, SIRT1 can exert an antifibrotic effect by acting on vascular endothelium by regulating Notch signaling and MMP-14 production (Vasko et al., 2014; Kida et al., 2016; Wei et al., 2017) and modulation of PGC-1 alpha (Han et al., 2017).

SIRT3 may possess antifibrotic properties due to its actions on mitochondria through weakening TGF-beta dependent signaling that could otherwise result in transforming renal fibroblasts into myofibroblasts, cells capable of synthesizing extracellular matrix (Sundaresan et al., 2015). In addition, SIRT3 activators, like honokiol, alleviate renal fibrosis in mice with unilateral ureter obstruction (Quan et al., 2020).

Increasing evidence suggests that aberrant activation of beta-catenin signaling is associated with the pathogenesis of fibrotic diseases, including renal fibrosis. Aberrant beta-catenin activation in mice was linked to increased expression of fibrosis-related proteins, such as fibronectin, matrix metalloproteinase 7, fibroblast-specific protein 1, plasminogen activator inhibitor 1, and Axis inhibition protein 2. SIRT6 may alleviate the course of obstructive nephropathy in mice due to transcriptional silencing of beta-catenin target genes through H3K56 DAC (Cai et al., 2020).

Antifibrotic actions of sirtuins, described above, can take part in delaying renal aging and age-related renal fibrosis (Sundaresan et al., 2015), as well as in mediating nephroprotective actions of angiotensin II receptor blockers (Kim et al., 2017). Antifibrotic actions of sirtuins may also mediate amelioration of the course of experimental obstructive nephropathy in mice (Quan et al., 2020).

### Direct Anti-aging Actions of Sirtuins in the Kidneys

SIRT1 (Braidy et al., 2011; Mitchell et al., 2014; Guan et al., 2017) and SIRT3 (Benigni et al., 2009; Redman and Ravussin, 2011) can directly slow down renal aging due to their actions on PGC-1 alpha/PPAR-gamma (prevention of metabolic syndrome and thus diabetic nephropathy), FoxO3 (activation, with subsequent induction of MnSOD and fall in the intracellular ROS concentration), FoxO4 (activation, with a subsequent promotion of removal of damaged or senescent cells through autophagy), and NF-κB (anti-inflammatory, and hence antifibrotic effect) (Kume et al., 2010; Zhang et al., 2016). Furthermore, aging of the kidneys can be slowed down both by NAD^+^ boosters (NMN) and by sirtuin-activating compounds (SRT 1720) (Mitchell et al., 2014; Chuang et al., 2017).

## Actions of Sirtuins in the Liver

### Anti-inflammatory Actions

SIRT1, SIRT3, SIRT4, and SIRT6 can exert anti-inflammatory actions in the liver (Ezhilarasan and Najimi, 2021). SIRT1 inhibits the activity of hepatic macrophages and inactivates the p65 subunit of NF-κB through deacetylation, desensitizing the liver to TNF-alpha (Chen et al., 2002; Yeung et al., 2004; Zhang et al., 2009). SIRT3 inhibits the production of pro-inflammatory chemokines and some pro-fibrotic factors (LoBianco et al., 2020). SIRT4 also exerts an anti-inflammatory effect since its deficiency may enhance inflammation, promote infiltration of macrophages, and develop hepatic cell carcinoma (HCC) (Li et al., 2019). The anti-inflammatory role of SIRT6 is based on a finding that its decreased activity worsens the course of alcohol-induced hepatitis in mice and promotes oxidative stress (Kim et al., 2019).

Unlike other sirtuins, SIRT2 may exert a pro-inflammatory effect since its deficiency can decrease thioacetamide-induced hepatic injury in mice through inhibition of NF-κB and MAPK dependent pro-inflammatory signaling (Jiao et al., 2019).

### Hepatic Steatosis Prevention by Sirtuins

SIRT1, SIRT3, and SIRT6 can oppose the development of fatty liver disease. SIRT1 decreases the expression of lipogenic enzymes, such as SREBP-1c, fatty acid synthase (FAS) and acetyl-coenzyme A carboxylase (ACC1) (Yamazaki et al., 2009). Both melatonin and SRT 1720 inhibit the development of non-alcoholic fatty liver disease (NAFLD) through indirect or direct activation of SIRT1 (Niu et al., 2018; Stacchiotti et al., 2019). In addition, SIRT1 deacetylates QKI-5 protein, which inhibits NAFLD development in mice, due to inhibition of triglyceride synthesis through modulation PPAR-gamma- and FoxO1-dependent signaling (Zhang et al., 2019). SIRT3 improves mitochondrial function and inhibits mitochondrial damage-induced hepatocyte apoptosis (Cho, 2014; Li R. et al., 2018). However, too high SIRT3 activity may worsen alcoholic liver disease through mitophagy inhibition (Ma et al., 2019). SIRT6 stimulates fatty acid oxidation through activation of PPAR-gamma; thus, SIRT6 deficiency may worsen the course of non-alcoholic steatohepatitis (NASH) in mice (Kim et al., 2010; Naiman et al., 2019; Zhong et al., 2020).

### Antifibrotic Actions of Sirtuins in Liver

SIRT1 and SIRT3 may counteract liver fibrosis. SIRT1 can exert this effect through activating PPAR-gamma and deacetylation of EZH2 protein. In liver fibrosis, SIRT1 may be transcriptionally repressed by HDAC4 (Li M. et al., 2017; Li et al., 2018). Other negative regulators of SIRT1 in the course of liver fibrosis can be micro-RNAs, such as miR-200a and miR-9a-5p (Qi et al., 2015; Yang J. J. et al., 2017).

Unlike SIRT1, SIRT2 may promote liver fibrosis because inhibition of its activity results in a decreased activity of other fibrosis-related proteins, such as alpha-SMA, COL1A1, MMP2, TIMP-1, and TIMP-2 (Kim et al., 2010).

The role of SIRT3 in liver fibrosis is less known, although SIRT3 activators may weaken the expression of fibrosis-related proteins, such as HMGB-1, type 1 collagen, and alpha-SMA (Wang et al., 2019).

### Other Actions of Sirtuins in the Liver

SIRT2 and SIRT3 decrease invasiveness of hepatic cell carcinoma (HCC) (Kim H. S. et al., 2011; Zhang B. et al., 2013; Zeng et al., 2017; Chen et al., 2019), while SIRT1, SIRT5, and SIRT7 may act as cytoprotective agents for HCC cells, so the therapeutic strategy in such case is their inhibition, not activation (Kim et al., 2013; Chang et al., 2018; Farcas et al., 2019; Wei et al., 2019; Zhao et al., 2019). As to SIRT6, research studies on its role in HCC have given inconsistent results (Feng et al., 2015; Ran et al., 2016; Huang et al., 2018).

In the course of hepatitis B, SIRT1, SIRT2, and SIRT6 seem to promote HBV replication (Ren et al., 2014; Deng et al., 2017; Yang Y. et al., 2017; Cheng et al., 2018), so in HBV infection, their therapeutic targeting should consist in inhibition, not activation (Yu et al., 2018; Jiang et al., 2019).

## The Role of Sirtuins in the Hematopoietic and Immune System

Hematopoietic stem cells (HSCs) in the bone marrow can undergo differentiation to any mature blood cells. HSCs can be divided into long-term HSCs (LT-HSCs), short-term HSCs (ST-HSCs), and multipotential progenitor precursors (MPPs), which may undergo both differentiation and proliferation, to maintain their number at a steady level. While ST-HSCs and MPPs provide everyday hematopoietic function, LT-HSCs do not proliferate in standard conditions. Still, they may become activated in case of hematopoietic stress – i.e., increased demand for hematopoiesis (Vazquez et al., 2021). Sirtuins are particularly important for maintaining proliferative quiescence of LT-HSCs, and thus their availability in case of hematopoietic stress.

As to the role of individual sirtuins in the regulation of hematopoiesis, they can be divided into two groups. The first one, consisting of SIRT6 and SIRT7, is especially significant for appropriate hematopoiesis in adults. The second one, consisting of SIRT1 and SIRT2, is significant from the standpoint of fetal hematopoiesis. SIRT6 KO and SIRT7 KO HSCs show some phenotypic traits typical for aged cells, i.e., abnormally high proliferation rate due to incapability to maintain mitotic quiescence (Mohrin et al., 2015; Wang et al., 2016). In addition, SIRT6 KO and SIRT7 KO mice show a progeroid phenotype, consisting of abnormally high HSCs in their bone marrow, combined with leukopenia found in their peripheral blood (Ou et al., 2011; Matsui et al., 2012; Mohrin et al., 2015; Vazquez et al., 2016; Wang et al., 2016). Both SIRT1 and SIRT2 deacetylate H4 histone at lysine 16 and thus remove epigenetic mark added by MOF protein – also an essential regulator of hematopoiesis (Vaquero et al., 2006).

In case of increased demand for hematopoiesis, some LT-HSCs leave the G1 phase of their cell cycle, thus allowing increased hematopoiesis; simultaneously, some of those cells proliferate to provide auto-replenishment. SIRT1 protects HSCs from DNA damage through deacetylation of FoxO3a, thus providing more effective DNA damage repair and promoting differentiation of adequately large percentage of HSCs into lymphocytes (Matsui et al., 2012; Singh et al., 2013; Rimmele et al., 2014). SIRT2 protects HSCs from programmed cell death through inhibition of NRLP3 inflammasome that may be otherwise activated as a result of mitochondrial dysfunction with subsequent oxidative damage (Luo et al., 2019). SIRT3 promotes mitochondrial homeostasis and well-being within HSCs, through its widely known actions within the mitochondria, such as improving overall ETC efficacy, induction of MnSOD, and thus mitigating oxidative stress (Brown et al., 2013). SIRT6 takes part in the maintenance of LT-HSCs metabolic quiescence by inhibiting Wnt signaling through H3K56 DAC, and thus transcriptional silencing of its target genes (Wang et al., 2016). HSC-protecting role of SIRT7 consists in the inhibition of mitochondrial protein synthesis in response to mitochondrial protein folding stress. In this way, SIRT7 prevents mitochondrial damage from unfolded protein response (Mohrin et al., 2015; Sigurdsson and Miharada, 2018). In adults, deficiency of SIRT1, SIRT6, and SIRT7 impairs HSC stress response. In addition, SIRT1 KO cells are more prone to DNA damage induced by cytostatics or irradiation (Singh et al., 2013; Rimmele et al., 2014).

SIRT6 KO HSCs have impaired capability of blood repopulation, mainly due to LT-HSCs deficiency appearing in such cases (Wang et al., 2016). SIRT6 maintains HSC number and functions at steady levels through deacetylation of H3 histone at lysine 56, promoting DNA damage response and transcriptionally inhibiting Wnt signaling (Wang et al., 2016). After a bone marrow transplant from SIRT7 KO donors, mice are more prone to graft damage with 5-fluorouracil and have an impaired peripheral blood regenerative capability than mice that have received a bone marrow transplant from wild type healthy donors (Mohrin et al., 2015; Vazquez et al., 2016). Besides, mitotically quiescent HSCs synthesize few proteins and thus have a lower risk of synthesizing unfolded or misfolded proteins that could initiate unfolded protein response (UPR) (Sigurdsson and Miharada, 2018). This *status quo* is supported by SIRT7, which maintains overall protein synthesis at a low level, thus preventing the production of unfolded or misfolded proteins (Mohrin et al., 2015).

Age-related dysfunction of the hematopoietic system results from cell damage accumulation which may cause anemia, adaptive immunity impairment, and increased incidence of myeloproliferative diseases. In aged HSCs, this damage is associated with epigenetic alterations, loss of cell polarity, DNA damage accumulation, and increased ROS production. In addition, the phenotypic traits mentioned above impair self-replenishment, balanced differentiation, and proliferation control of HSCs (Brown et al., 2013; Mohrin et al., 2015; Vazquez et al., 2021).

SIRT1, SIRT2, SIRT3, and SIRT7 expression becomes decreased with age, which impairs HSC homeostasis (Brown et al., 2013; Rimmele et al., 2014; Mohrin et al., 2015; Luo et al., 2019). SIRT1 KO young HSCs resembled aged cells as to the pattern of transcription (Rimmele et al., 2014), while SIRT2 and SIRT3 seem necessary to maintain completely functional HSCs in old mice, but not for adaptation to hematopoietic stress in young mice (Brown et al., 2013; Roth et al., 2013). SIRT2 can prevent deterioration of HSC function with age through activation of NRLP3 inflammasome in response to mitochondrial damage (Luo et al., 2019), while SIRT3 expression in HSCs is pronounced but falls with age. SIRT3 depletion does not impair the blood repopulation capability in mice, but – in experiments with serial bone marrow transplants in mice – SIRT3 is necessary for bone marrow regeneration, from the third transplant on (Brown et al., 2013).

In addition, SIRT1 deficiency may result in a tendency of HSCs to differentiate more into granulocytes than into lymphocytes (Rimmele et al., 2014; Abraham et al., 2019). SIRT6 and SIRT7 deficiency may have a similar result. Deficiency of SIRT6 results in an abnormally high percentage of MPPs in the bone marrow and peripheral blood. In the case of SIRT7 deficiency, abnormally high percentage of MPPs is found only in peripheral blood (Mohrin et al., 2015; Lasiglie et al., 2016; Wang et al., 2016). SIRT2 KO mice show a similar abnormality, but only in old age (Luo et al., 2019). As expected, increased expression of SIRT2, SIRT3, and SIRT7 can neutralize this abnormality (Brown et al., 2013; Mohrin et al., 2015; Luo et al., 2019).

In B lymphocytes, sirtuins increase their vitality, inhibit apoptosis (SIRT1), and act as tumor suppressor proteins (SIRT3 and SIRT4). As to the effect of sirtuin activity on T cells, decreased activity of SIRT1 in Th and Tc cells results in a tendency to their hyperactivation, but the reduced activity of SIRT3 in Treg cells can impair their function. Probably this is why a high-calorie diet promotes autoinflammatory and autoimmune response, while caloric restriction may diminish the efficacy of adaptive immune response, thus being contraindicated in severe infections (Warren and MacIver, 2019).

## Sirtuins in the Prevention of Genomic Instability and Unrepaired DNA Damage

SIRT1 induction promotes genomic stability (Herranz et al., 2010) because SIRT1 stimulates DNA damage repair and can co-activate another sirtuin – SIRT6, which in turn deacetylates the H3 histone at Lys 56, promoting DNA repair and conservation through silencing gene expression. In addition, both SIRT1 and SIRT6 can activate p53, despite inhibiting its proapoptotic activity. In general, nuclear sirtuins can promote DNA conservation through histone deacetylation/deacylation and transcriptional silencing (Guarente, 2000; Haigis and Sinclair, 2010). In contrast, mitochondrial sirtuins can prevent DNA damage through abrogating ROS production (SIRT3) (Huang et al., 2010; Haigis et al., 2012), or even supporting nucleotide synthesis, necessary for DNA repair, through glutamine anaplerosis (SIRT4) (Jeong et al., 2013). By deacetylating histones, SIRT1 and SIRT6 can promote genome stability, silence the “transcriptional noise” that is a threat to old cells, and minimize the risk of DNA damage, since DNA bound to deacetylated or deacylated histones is less accessible both for transcription factors and for mutagens (Michishita et al., 2009; Yang et al., 2009; Jiang et al., 2013).

By activating some DNA-repairing enzymes, such as WRN helicases, DNA-PKcs, CtIP, and PARP-1, SIRT6 promotes DNA damage repair, including repair of double-strand breaks (DSB) through homologous recombination (HR). Due to this function, SIRT6 can prevent both carcinogenesis and aging, because both of these phenomena depend primarily on the accumulation of unrepaired DNA damage (Huertas, 2010; Beneke, 2012).

SIRT6 KO cells have shown overexpression of subtelomeric genes, such as ISG-16, which can accelerate telomere attrition. Thus, active SIRT6 can protect cells also from replicative stress (Mao et al., 2011; Tennen et al., 2011).

SIRT6 inhibits HIF-1 alpha and HIF-1 alpha-dependent glucose transport to the cells, relying on glucose transporters GLUT1 and GLUT4 (Seagroves et al., 2001; Xiao et al., 2010). Whereas Otto Warburg has already described the harmful effects of HIF-1 alpha hyperactivation in neoplastic cells in 1956 (Warburg, 1956), SIRT6 prevents hyperactivation of HIF-1 alpha in normal cells, thus preventing lethal hypoglycemia due to glucose uptake by too many cells at the same time (Xiao et al., 2010).

SIRT6 actions toward other aspects of cell phenotype (including inhibition of c-Jun, and thus IIS, rescuing p53 function, and activation of CCNDBP-1) can prevent carcinogenesis. Therefore, SIRT6 can be regarded as a TSP because of being a caretaker itself and its ability to activate many other caretakers – like DNA-repairing enzymes and gatekeepers – like p53 (Michishita et al., 2008; Das et al., 2011; Sundaresan et al., 2012; Wątroba and Szukiewicz, 2021).

## Conclusion

Sirtuins may counteract organismal aging by altering the pattern of cellular stress response to generate much less disruption of tissue homeostasis. The alteration of cellular stress response pattern by sirtuins comprises 1- inhibition of apoptosis, 2- promoting DNA damage repair instead of apoptosis or CSP induction, 3- antioxidative action through activation of MnSOD, 4- preventing carcinogenesis through acting as TSPs, 5- inhibition of unnecessary inflammatory response/inflammaging through inactivation of NF-kB, and 6- preventing CSP and senescence-associated secretory phenotype (SASP) through mitochondrial protection and promoting DNA damage repair.

All the effects listed above combined may prevent disruption of tissue homeostasis – directly responsible for organismal aging in vertebrates while being itself a distant derivative of a prolonged, inappropriate pattern of cellular response to accidental damage of the biostructure. The mechanisms discussed in this article describe how exactly sirtuin-dependent modifications of the cellular stress response can slow down aging at the tissue level. Thus, sirtuins, especially SIRT1, SIRT3 and SIRT6, can modify cellular stress response to promote maintenance of tissue homeostasis and thus slow down phenotypic aging at the organismal level.

## Author Contributions

MW has made a critical review of the literature and wrote the revised version of the manuscript. DS contributed to the overall conception of this review including design and content of the figures and wrote the first draft of the manuscript. Both authors contributed to manuscript revision, read and approved the submitted version.

## Conflict of Interest

The authors declare that the research was conducted in the absence of any commercial or financial relationships that could be construed as a potential conflict of interest.

## Publisher’s Note

All claims expressed in this article are solely those of the authors and do not necessarily represent those of their affiliated organizations, or those of the publisher, the editors and the reviewers. Any product that may be evaluated in this article, or claim that may be made by its manufacturer, is not guaranteed or endorsed by the publisher.
